# Columnar Lesions in Barrel Cortex Persistently Degrade Object Location Discrimination Performance

**DOI:** 10.1523/ENEURO.0393-22.2022

**Published:** 2022-11-11

**Authors:** Lauren Ryan, Maya Laughton, Andrew Sun-Yan, Samantha Costello, Ravi Pancholi, Simon Peron

**Affiliations:** Center for Neural Science, New York University, New York, New York 10003

**Keywords:** barrel cortex, lesion, perception

## Abstract

Primary sensory cortices display functional topography, suggesting that even small cortical volumes may underpin perception of specific stimuli. Traditional loss-of-function approaches have a relatively large radius of effect (>1 mm), and few studies track recovery following loss-of-function perturbations. Consequently, the behavioral necessity of smaller cortical volumes remains unclear. In the mouse primary vibrissal somatosensory cortex (vS1), “barrels” with a radius of ∼150 μm receive input predominantly from a single whisker, partitioning vS1 into a topographic map of well defined columns. Here, we train animals implanted with a cranial window over vS1 to perform single-whisker perceptual tasks. We then use high-power laser exposure centered on the barrel representing the spared whisker to produce lesions with a typical volume of one to two barrels. These columnar-scale lesions impair performance in an object location discrimination task for multiple days without disrupting vibrissal kinematics. Animals with degraded location discrimination performance can immediately perform a whisker touch detection task with high accuracy. Animals trained *de novo* on both simple and complex whisker touch detection tasks showed no permanent behavioral deficits following columnar-scale lesions. Thus, columnar-scale lesions permanently degrade performance in object location discrimination tasks.

## Significance Statement

Sensory cortical inactivation perturbs many behaviors. Whether small patches of sensory cortex are necessary for behavior remains unclear as typical inactivation methods have poor resolution (>1 mm), and few studies allow for behavioral recovery. In the rodent whisker system, individual whiskers map to cortical “barrels” with a radius of ∼150 μm. We produce lesions on the scale of one to two barrels. We find that such lesions permanently degrade performance in tasks that require mice to discriminate between object locations using their whiskers, but not in tasks that require mice to report the presence or absence of an object. Thus, volumes of somatosensory cortex containing around 10,000 neurons act as perceptual bottlenecks in object location discrimination but not object detection behaviors.

## Introduction

To understand the neural basis of perception, it is important to identify brain areas that causally contribute to behavior. The behavioral necessity of a brain area is typically assessed using loss-of-function perturbations. Across sensory modalities, such experiments suggest that primary sensory cortices are essential for some tasks but not others (Lashley, 1931a,b; Schneider, 1969; Hutson and Masterton, 1986; Newsome and Paré, 1988; Prusky and Douglas, 2004; Glickfeld et al., 2013; Shih et al., 2013; Poort et al., 2015; Goard et al., 2016; Resulaj et al., 2018; Stüttgen and Schwarz, 2018; Slonina et al., 2022). Typical loss-of-function experiments inactivate spatially extensive tissue volumes and rarely allow for behavioral recovery, complicating interpretation and leaving open the question of whether small cortical volumes act as permanent perceptual bottlenecks.

Traditional loss-of-function perturbations have poor spatial resolution: permanent lesions (Lashley, 1931a; Hutson and Masterton, 1986; Resulaj et al., 2018) typically have a radius in excess of 1 mm, impacting hundreds of thousands of neurons. Transient loss-of-function experiments require careful calibration to measure effect radius. Unfortunately, this is rarely done, and when it is, the radius of effect is found to often be in excess of 1 mm: optogenetic silencing drives inactivation >1 mm away even when the spatial extent of stimulation light is far smaller (Li et al., 2019); pharmacological inactivation requires careful calibration to achieve a radius of effect of ∼1 mm (Krupa et al., 1999); Peltier cooling exerts effects at least 1 mm away (Long and Fee, 2008). In addition, most loss-of-function studies do not allow time for behavioral recovery, leaving open the question of whether other structures could compensate for the experimentally induced loss. Indeed, lesions of brain areas sensitive to transient inactivation reveal that behavioral recovery can occur relatively quickly, and it is often unclear whether sensitivity to transient inactivation is because of method-specific downstream effects (Otchy et al., 2015; Hong et al., 2018; Wolff and Ölveczky, 2018). Thus, more spatially focused loss-of-function approaches combined with long-term monitoring of behavior are essential for identifying the minimal subset of cortical activity needed for specific behaviors.

In the mouse vibrissal system, individual whiskers project to small, defined patches of primary vibrissal somatosensory cortex (vS1) called “barrels” (radius, ∼150 μm; Lefort et al., 2009). Most inactivation studies in vS1 do not explore the behavioral contribution of individual barrels because of poor spatial resolution. For instance, optogenetic inactivation experiments targeting vS1 often directly perturb activity not just in vS1 but also in adjacent structures such as secondary vibrissal somatosensory cortex. Lesions of vS1 are also usually extensive, often impacting adjacent cortical areas along with subcortical structures (Hutson and Masterton, 1986; Hong et al., 2018). Inactivation of vS1 degrades performance on aperture size discrimination (Krupa et al., 2001), object location discrimination (O’Connor et al., 2010; Guo et al., 2014b), gap crossing (Hutson and Masterton, 1986; Shih et al., 2013), texture discrimination (Guic-Robles et al., 1992), and whisker touch detection (Miyashita and Feldman, 2013; Sachidhanandam et al., 2013; Hong et al., 2018) tasks. However, whether vS1 is necessary for these behaviors remains unclear: mice can relearn a touch detection task in a single day following vS1 aspiration, but transient optogenetic inactivation degrades performance (Hong et al., 2018). While vS1 is thus implicated in many tasks, in most cases, it remains unclear whether perceptually relevant activity is mostly confined to the barrels of specific whiskers and whether observed behavioral deficits are permanent.

Here, we precisely lesion small volumes of barrel cortex in awake mice previously implanted with a cranial window using a femtosecond laser. Our approach obviates the need for postoperative recovery and allows for the lesioning of a targeted barrel along with partial removal of adjacent barrels. We perform lesions immediately before behavioral testing, and track performance for several days to allow for behavioral recovery. In mice performing a vibrissal go/no-go object location discrimination task with a single whisker, lesions centered around the barrel representing the spared whisker persistently degraded performance for several days. Lesions did not impact whisking kinematics. Mice trained on a go/no-go object detection task showed a small, transient decline in performance after a columnar-scale lesion in vS1, suggesting that such tasks are not dependent on individual vS1 barrels. Mice trained on a more complex detection task with two response contingencies and a delay period between stimulus presentation and response also showed only a small and transient decline in performance after columnar-scale lesions, suggesting that even this more complex detection task ultimately does not require individual barrels. Thus, we find that columnar-scale vS1 lesions result in permanent performance degradation on vibrissal object location discrimination but not touch detection tasks.

## Materials and Methods

### Animals

Adult C57BL/6J (stock #000664, The Jackson Laboratory) mice (9 female, 13 male) and Ai162 (stock #031562, The Jackson Laboratory) × Slc17a7-Cre (stock #023527, The Jackson Laboratory; Daigle et al., 2018) mice (4 female, 3 male) were used (Table 1). In cortex, Ai162 × Slc17a7-Cre mice express GCaMP6s in excitatory neurons. To suppress transgene expression during development, Ai162 × Slc17a7-Cre breeders were fed a diet that included doxycycline (625 mg/kg doxycycline; Teklad), so that mice received doxycycline until weaning. All animal procedures were approved by the New York University Animal Welfare Committee.

**Table 1:** List of animals

Animal ID	Genotype	Task	Sex	Lesion volume (mm^3^)	Whisker	Behavior start age (weeks)
014712*	C57BL/6J	Go/no-go location discrimination	F	1.3214	C2	11
014377	C57BL/6J	Go/no-go location discrimination	M	0.1891	C2	9
014390	C57BL/6J	Go/no-go location discrimination	M	0.2256	C2	8
014389	C57BL/6J	Go/no-go location discrimination	M	0.1483	C2	9
014388	C57BL/6J	Go/no-go location discrimination	M	0.0494	C2	9
014378*	C57BL/6J	Go/no-go location discrimination	F	1.0068	C3	9
009848	C57BL/6J	Go/no-go location discrimination	F	0.3156	C2	16
009840	C57BL/6J	Go/no-go location discrimination	M	0.162	C2	10
012266	C57BL/6J	Go/no-go location discrimination	M	0.0996	C2	9
296116**	slc17a7-Cre × Ai162	Go/no-go location discrimination	M	0.1717	C2	15
283529*	slc17a7-Cre × Ai162	Go/no-go detection	M	0.9621	C2	12
283541*	slc17a7-Cre × Ai162	Go/no-go detection	F	0.5002	C2	12
293169	slc17a7-Cre × Ai162	Go/no-go detection	F	0.5008	C3	16
293317	slc17a7-Cre × Ai162	Go/no-go detection	F	0.1405	C2	16
293158	slc17a7-Cre × Ai162	Go/no-go detection	M	0.2883	C2	18
295141***	slc17a7-Cre × Ai162	Go/no-go detection	F	0.7130†	C3	9
014324****	C57BL/6J	Go/no-go detection	M	0.0836	C2	11
009850	C57BL/6J	Go/no-go detection	M	0.1035	C2	12
014356	C57BL/6J	Go/no-go detection	F	0.0887	C2	15
014357	C57BL/6J	Go/no-go detection	F	0.07274†	C2	16
014386	C57BL/6J	2 lickport detection	M	0.0655†	C2	21
014174*	C57BL/6J	2 lickport detection	F	1.3214	C2	11
014173*	C57BL/6J	2 lickport detection	M	0.0321	C2	11
014385	C57BL/6J	2 lickport detection	M	0.1757†	C3	11
014374	C57BL/6J	2 lickport detection	F	0.1286	C2	11
014366****	C57BL/6J	2 lickport detection	F	0.0830†	C2	11
014364	C57BL/6J	2 lickport detection	F	0.0710	C2	11
009849	C57BL/6J	2 lickport detection	M	0.0936	C2	10
017749	C57BL/6J	2 lickport detection	M	0.1109	C2	10

Mice are divided into task-based cohorts. Symbols for individual mice are unique and consistent throughout the article within a cohort. F, Female; M, male.

*Mice with excessively large or small lesions, excluded from post lesion analyses but included in pre lesion analyses. **Mice with no whisker videography, excluded from lesion and video-dependent analyses. ***Mouse that showed unusually high ignore rate post lesion, and so was excluded from post-trim analysis. ****Mice with poor slice quality, included in analysis, but the representative slice not shown. †Lesion size estimated because of missing slices (Materials and Methods).

### Surgery

Mice (6–10 weeks old) were anesthetized with isoflurane during cranial window and headbar implantation (3% induction, 1.5% maintenance). A titanium headbar was attached to the skull with cyanoacrylate (Vetbond). A circular craniotomy (diameter, 3.5 mm) was made in the left hemisphere over vS1 (center: 3.5 mm lateral, 1.5 mm posterior from bregma) using a dental drill (FG 1/4 drill bit, Midwest Tradition). Following the craniotomy, a double-layer cranial window (external diameter, 4.5 mm; inner diameter, 3.5 mm; #1.5 coverslip; adhered with Optical Adhesive 61, Norland Products) was placed over the craniotomy. The cranial window and headbar were affixed to the skull with dental acrylic (Orthojet, Lang Dental).

### Behavior

Following surgical recovery, mice were water restricted and trimmed to whiskers C1 to C3. Mice were trained on one of the following three tasks. (1) Go/no-go object location discrimination task. A metal pole (diameter, 0.5 mm; Drummund Scientific) moved vertically (i.e., along the dorsal–ventral axis of the animal) into the mouse’s whisking plane in a series of proximal positions or in a series of distal positions. Mice were rewarded with water for licking while the pole was in reach if the pole appeared in one of the proximal positions (in reach time, 2–3 s). Licking when the pole appeared in one of the distal positions led to a timeout period and an aversive sound. (2) Go/no-go object detection task. A metal pole moved vertically into the mouse’s whisking plane in or out of reach of its one spared whisker. Mice were rewarded for licking when the pole was in reach and given a timeout period and an aversive sound for licking when the pole was out of reach. (3) Lick-left/lick-right object detection task. Each trial consisted of three epochs: stimulus epoch (2 s); delay (300 ms); and response (<2 s). During the stimulus epoch, a metal pole moved vertically into the whisking plane of the mouse at an in-reach proximal position or an out-of-reach distal position and then moved outside the whisking plane. During the delay epoch, the lickport moved into reach. During the response epoch, an auditory cue indicated the mouse should respond, and mice were rewarded for licking the right lickport during in reach trials, and the left lickport during out-of-reach trials. Incorrect responses resulted in a timeout period and immediate withdrawal of the lickport.

All behavioral training proceeded in a standard sequence. First, mice were handled and habituated to the behavioral apparatus. After habituation, mice were trained to lick for a water reward and then proceeded directly to behavioral training on the go/no-go tasks. For the two-lickport task, mice also learned the timing of the task and to lick both left and right lickports before training progressed by using blocks where only a single lickport was rewarded. In the location discrimination task, mice started with distal positions out of reach. Distal positions were quickly brought into reach once mice became accustomed to the task structure and whisked vigorously. In detection tasks, the pole was presented in a position that was easily reached but did require active whisking. All animals began training on dedicated training rigs. Once animals learned the task using whiskers C1 to C3 (i.e., attained a d′ > 1.5), animals were trimmed to a single whisker (typically C2, occasionally C3; Table 1). Subsequent trimming occurred every 2–3 d. To collect whisker video during behavior, mice were moved to a rig with whisker videography once they were proficient at the final version of their task. Lesions in these animals were not performed until they exhibited stable performance on the whisker videography rig, as some animals took one to two sessions to adjust to the new apparatus.

The behavioral task was controlled by a BPod State Machine (Sanworks) and custom MATLAB software (MathWorks) running on a behavioral computer (System 76). The auditory response tone was controlled by a low-latency audio board (Bela). Lickport motion was controlled by a set of three motorized actuators (Zaber) and an Arduino. Licks were sensed using a custom electrical detection circuit.

### Barrel identification

For the Ai162 × Slc17a7-Cre mice, the locations of barrels in vS1 corresponding to whiskers C1 to C3 were identified by measuring the GCaMP6s Δ*F*/*F* at coarse resolution (4×; field of view, 2.2 × 2.2 mm) on a two-photon microscope while the whiskers were individually deflected with the standard stimulus pole. This was done in awake mice not engaged in a task. Barrel locations in the C57BL/6J mice were identified using intrinsic signal imaging while the whiskers were individually deflected. In this case, mice previously implanted with vS1 cranial windows were anesthetized with isoflurane (typically, 1%). Imaging was performed with a 10 bit ACE camera (Basler) mounted on a stereoscope and using custom MATLAB software. Green illumination (catalog #M530L4, Thorlabs) was used to image the vasculature, and red illumination (catalog #M625L4, Thorlabs) was used for functional imaging. Individual whiskers were placed inside a capillary tube and stimulated with a piezo stimulator (catalog #PB4NB2S, Thorlabs). Stimulation consisted of 5 repetitions of 10 deflections, each lasting 50 ms, with a 100 ms interval in between. Deflections typically resulted in an angular displacement of 5–10° at the follicle. For each whisker, the region with the highest change in reflectance (ΔR/R) was mapped with respect to the vasculature, and two to three whiskers were mapped per mouse.

### Whisker videography

Whisker video was acquired using custom MATLAB software from a CMOS (complementary metal–oxide–semiconductor) camera (model Ace-Python 500, Basler) running at 400 Hz and 640 × 352 pixels and using a telecentric lens (model TitanTL, Edmund Optics). Illumination was provided by a pulsed 940 nm LED (model SL162, Advanced Illumination) operating in synchrony with the camera (typical exposure and illumination duration, 200 μs). Seven to nine seconds of each trial were recorded, including 1 s before pole movement, the period when the pole was in reach, and several seconds after the pole was retracted. Data were processed at the High Performance Computing cluster at New York University. First, candidate whiskers were detected using the Janelia Whisker Tracker (Clack et al., 2012). Next, whisker identity was refined and assessed across a single session using custom MATLAB software (Peron et al., 2015, 2020). Following whisker assignment, whisker curvature (κ) and angle (θ) were calculated at specific locations along the length of the whisker.

Change in curvature, Δκ, was calculated relative to a resting angle-dependent baseline curvature value obtained during periods when the pole was out of reach. Next, automatic touch detection was performed. Touch assignment was manually curated using a custom MATLAB user interface (Peron et al., 2015). As per convention, protractions were assigned negative Δκ values. The angle (θ) was decomposed into whisking setpoint, amplitude, and phase via the Hilbert transform (Kleinfeld and Deschênes, 2011).

### Lesions

Cortical lesions were performed using a 1040 nm laser (Fidelity HP, Coherent) focused at a depth of 200–300 μm for 10–20 s at 1–1.5 W power. Lesions were either centered on the target barrel (experimental lesions) or in visual cortex (posterior and medial relative to the target barrel; sham lesions). Lesions targeted a single site. In Ai162 × Slc17a7-Cre mice, the desired barrel was found using coarse resolution two-photon imaging (4×), and lesion success was visually confirmed by an increase in GCaMP6s fluorescence in the target area. In C57BL/6J mice, the target barrel was found using epifluorescence imaging of the vasculature and the intrinsic imaging map as a reference. Lesion efficacy was often validated by performing an identical lesion in an Ai162 × Slc17a7 mouse immediately before lesioning of trained C57BL/6J mice. Because these mice expressed GCaMP6s, lesion extent could be calibrated based on the post lesion fluorescence. Animals were awake and head fixed in the behavioral apparatus during lesioning and were monitored for signs of distress or discomfort. Most animals were lesioned 5–30 min before the start of behavior, although a few animals were lesioned in the middle of a behavioral session.

Following behavioral training and lesioning, all animals were perfused with paraformaldehyde (4% in PBS) and postfixed overnight. Coronal sections 70–80 μm thick were cut on a vibratome (Leica) and mounted on glass slides with VECTASHIELD antifade mounting media containing DAPI (Vector Laboratories). Sections were imaged using a fluorescent light microscope (model VS120, Olympus).

To quantify lesion volume, we used DAPI (C57BL/6J mice) or GCAMP6s fluorescence (Ai162 × Slc17a7-Cre mice). Serial images of the lesion were collected using a fluorescent light microscope (model VS120, Olympus). All images in which a lesion was present were registered to the Allen Mouse Brain Common Coordinate framework using the SHARP-Track (Slice Histology Alignment, Registration, and Probe-Track) pipeline (Shamash et al., 2018). In this way, lesions from each animal were normalized to the standard brain atlas and lesions could be compared across animals. Lesion volume was quantified by measuring the area of manually delimited lesion borders across adjacent sections and calculating the volume as follows: *V* = (*A*_1_ + *A*_2_ + … + *A_n_*) * *t*, where *A_n_* is the lesion area in the final slice containing a lesion, and *t* is the thickness at which sections were sliced (Shih et al., 2013). For animals where some lesion slices were not recoverable, we estimated the lesion volume from the available slices. The slice with the largest lesion area was taken to be the maximum area the lesion reached across all slices. We linearly fit known volumes and maximal area across the animals for which all slices were present and used this fit along with the maximal area to estimate lesion volume. Animals for which volume was estimated in this manner are denoted with a dagger symbol in Table 1.

### Immunohistochemistry

Lesions were performed as described previously in two Ai162 × Slc17a7-Cre mice. Perfusion was performed 24 h after lesioning. The 50-μm-thick sections were then cut on a vibratome (Leica), and the sections that included the lesion were incubated overnight with primary antibodies made in 1% bovine serum albumin and 0.05% sodium azide under continuous agitation. Alternating slices were labeled for Iba1 and glial fibrillary acidic protein (GFAP; Podgorski and Ranganathan, 2016). Slices were washed three times and then incubated in a secondary antibody (1:500) conjugated to Alexa Fluor 647. Slices were rinsed and mounted using antifade mounting media (Vector Laboratories). Slices were imaged using a microscope (model VS120, Olympus) and a confocal microscope (model SP5, Leica).

The following primary antibodies were used: rabbit anti-Iba1 (1:500 dilution; catalog #019–19 741, Wako), mouse monoclonal anti-GFAP (1:1000 dilution; catalog #G3893, Sigma-Aldrich). The following secondary antibodies used were used: goat anti-mouse, Alexa Fluor 647 (Iba1 and HSP; catalog #A-21244, Thermo Fisher Scientific); and goat anti-rabbit, Alexa Fluor 647 (GFAP; catalog #A-21235, Thermo Fisher Scientific).

### Experimental design and statistical analyses

For comparisons between distinct samples, two-tailed unpaired *t* tests were used. For longitudinal comparisons within the same animals, paired *t* tests were used. For correlation tests, a Pearson’s correlation was used to identify a linear correlation coefficient (*R*) and test for significance. Ranges indicate mean ± SEM, unless indicated otherwise. All statistical analyses were performed using MATLAB. An exact list of animals used for each experiment is provided in Table 1.

## Results

### Mice use touch to solve a single whisker go/no-go object location discrimination task

**Figure 1.**
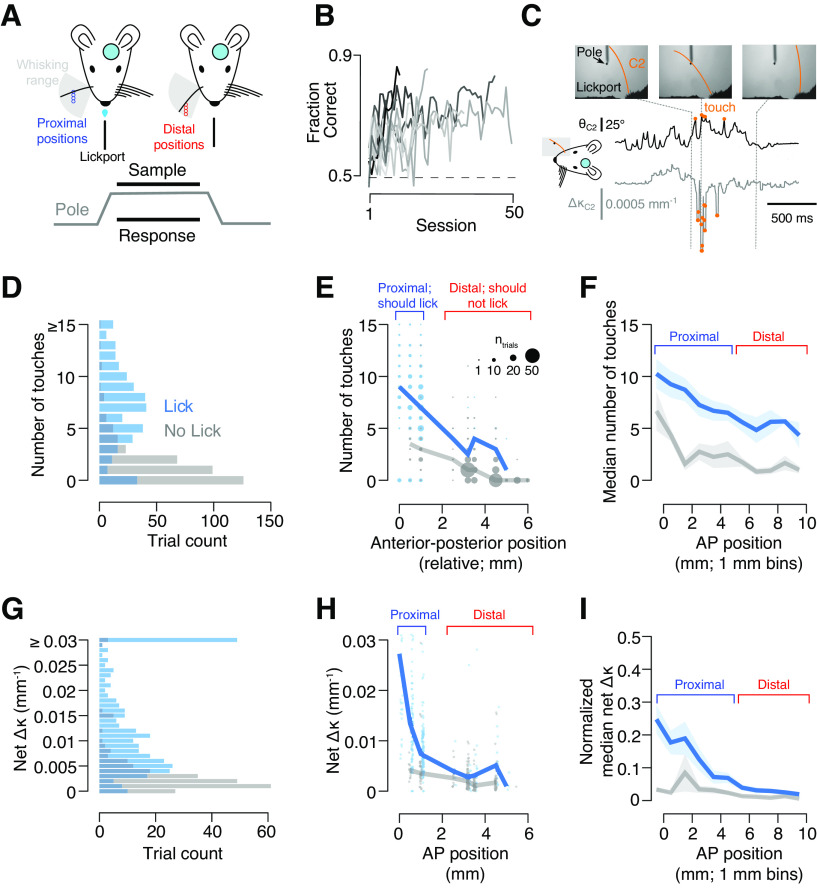
Mice use touch to solve a go/no-go object location discrimination task. ***A***, Task schematic. Top, Head-fixed mice with a cranial window over vS1 use a single whisker to localize a pole that appears in either posterior (blue) or anterior (red) positions. Bottom, Task timing. The pole is accessible during the sample period (1–2 s), and mice must respond by licking during this period. ***B***, Training progression for all go/no-go location discrimination mice (*n* = 10). ***C***, Whisker videography in an example trial. Top, Example frames. Bottom, Whisker angle (θ, black) and change in whisker curvature (Δκ, gray; see Materials and Methods), with touches overlaid as orange circles. ***D***, Number of trials with a specific touch count for the two pre lesion behavioral sessions in an example mouse. Light blue, Trials where the mouse made a lick response; gray, trials with no lick. ***E***, Number of touches as a function of pole position during the two pre lesion sessions for an example mouse. Thick lines, The mean for a position. Individual dots are sized to show how many trials with a given outcome, touch count, and position occurred. ***F***, Number of touches across anterior–posterior pole positions for all mice averaged across the two pre lesion sessions. Line and shaded region show mean and SEM (*n* = 9 mice). Positions across animals were aligned so that the transition between trials on which the mouse should and should not lick was at 5 mm. ***G***, Number of trials with a specific touch intensity (net Δκ) across two pre lesion behavioral sessions in an example mouse. ***H***, Touch intensity (net Δκ) as a function of pole position, for an example mouse in an example session. Thick lines, The median for a position. Individual dots show individual trials and are slightly jittered along the *x*-axis to facilitate visibility. ***I***, Touch intensity (median net Δκ, normalized to within-animal 99th percentile) across anterior–posterior pole positions for all mice. Line and shaded region show mean and SEM (*n* = 9 mice). Positions across animals were aligned as in ***F***.

We trained water-restricted mice implanted with a cranial window over vS1 to perform a head-fixed go/no-go object location discrimination task using a single whisker (Fig. 1*A*; Materials and Methods). Mice received a water reward for licking when a vertical pole was presented in a series of proximal positions (“go” trials). On “no-go” trials, the pole appeared in a series of distal positions and licking responses resulted in a timeout. Mice became proficient at this task in 12.9 ± 2.0 d (mean ± SD; *n* = 10 mice; Fig. 1*B*).

Once mice were proficient at the task, we used high-speed videography to assess the impact of vibrissal-object touch on behavior (Materials and Methods; Fig. 1*C*). Mice were sensitive to the number of object touches, licking more often on trials with a higher number of touches (Fig. 1*D*). The number of touches was position dependent, with anterior positions typically eliciting fewer touches (Fig. 1*E*,*F*). Across all pole positions, a higher number of touches was more likely to elicit a lick response.

Vibrissal S1 touch neurons show increasing responses to higher contact forces (Peron et al., 2015). We therefore examined the impact of touch force on behavior, using the net whisker curvature change on each trial as a proxy for force acting on the whisker follicle (Birdwell et al., 2007; Pammer et al., 2013). Force on the whisker follicle drives primary sensory afferent responses (Severson et al., 2017). Mice licked more frequently on trials with greater contact forces (Fig. 1*G*). At more posterior positions, contact force was higher, and, for any given pole position, higher contact force increased the likelihood of licking (Fig. 1*H*,*I*). Thus, both the frequency and intensity of touch impacted behavior in our task.

**Figure 2.**
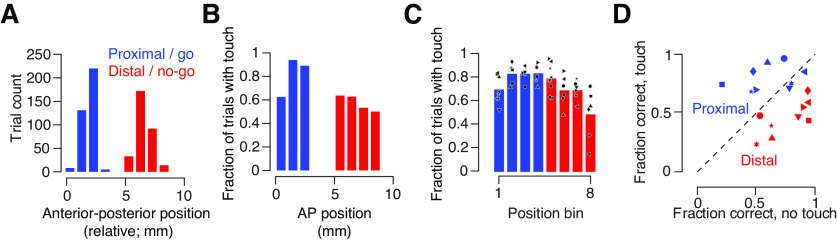
Mice touch reliably at both proximal and distal pole positions. ***A***, Number of trials at each pole position across the two pre lesion days in an example mouse. ***B***, Fraction of trials with touch on any given position for the same days as in ***B***. ***C***, Touch frequency across all pre lesion behavioral sessions with whisker videography (Materials and Methods) across all mice (*n* = 9). Positions were normalized across mice by binning both proximal and distal position ranges into four equal-sized bins. Symbols, Individual mice; bar, mean across mice. ***D***, Modulation of performance by touch during two pre lesion sessions. Symbols, Individual mice; blue, go/proximal trials; red, no-go/distal trials.

Mice often exhibit vibrissal foveation in object localization tasks, restricting whisker movement to the proximal positions, rather than whisking through their full whisking extent (O’Connor et al., 2010). This transforms the behavior from a location discrimination task that likely requires knowledge of whisker position and sensorimotor integration to a task where the animal simply must lick on any detected touch. To counteract this tendency, we used a series of proximal and distal pole positions and presented these positions at different frequencies tailored to individual mice (Fig. 2*A*). This resulted in a high number of object contacts at both proximal and distal pole positions (Fig. 2*B*,*C*). Most mice showed performance decrements on proximal trials where no touch occurred, although a few mice showed equal performance for proximal pole positions on touch and nontouch trials (Fig. 2*D*). Mice consistently performed worse on distal trials where touch occurred compared with distal trials without touch. Therefore, mice naturally tend toward a detection strategy, but our multiposition approach ensures that they continue to touch across both position ranges, necessitating that they perform an object location discrimination task.

### Columnar-scale vS1 lesions via prolonged femtosecond laser exposure

**Figure 3.**
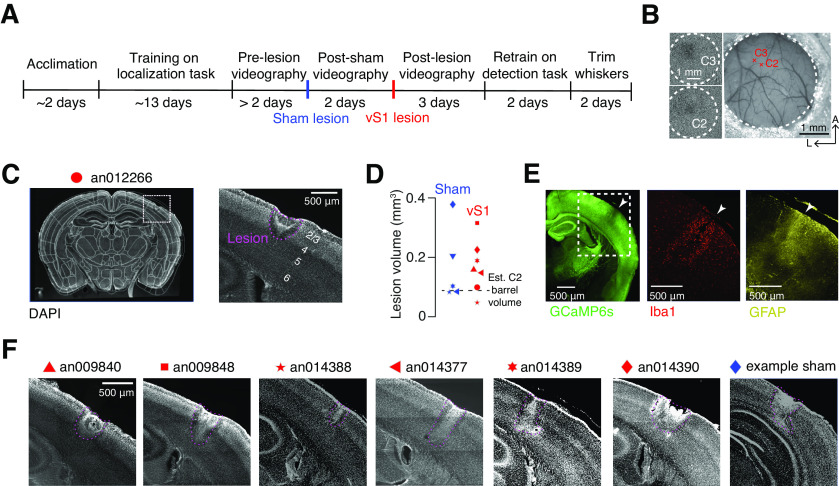
Columnar-scale vS1 lesioning approach. ***A***, Timeline of typical experiment. ***B***, Identification of barrels. Left, Intrinsic signal imaging response to individual deflection of the C2 or C3 whiskers. Dotted circle, Window. Right, Center of intrinsic response mapped onto the vasculature (Materials and Methods). ***C***, Post lesion histology. Example DAPI coronal section in mouse with a lesion. Left, Section registered to the Allen Brain Atlas. Right, Area around lesion, with lesion outlined in pink. ***D***, Sham lesion (blue) and vS1 lesion (red) volumes. Symbols, Individual mice; dashed line, estimated C2 barrel volume. ***E***, Post lesion immunohistochemistry in an example mouse. Left, GCaMP6s; middle, Iba1, a microglial marker; right, GFAP, an astrocytic marker. ***F***, Representative slice showing the lesion from all location discrimination animals and one example sham animal. Symbols correspond to ***D***. Wherever possible, the slice shown represents the widest observed lesion extent.

To determine whether individual vS1 columns are necessary for object location discrimination, we lesioned a target barrel along with portions of adjacent barrels in well trained animals and tracked performance for several days following the lesion (Fig. 3*A*; Materials and Methods). Barrels were first identified using intrinsic signal imaging (Fig. 3*B*), and their locations were mapped onto the vasculature (Materials and Methods). Lesions were performed by subjecting the target barrel to prolonged femtosecond laser exposure immediately before the first post lesion behavioral session. Lesions did not require any surgery or anesthesia as they were performed on mice previously implanted with cranial windows. Lesion volumes were comparable to the volume of two barrels: 0.17 ± 0.09 mm^3^ (mean ± SEM; *n* = 7 mice; volume of a single barrel: 0.09 mm^3^, assuming a ∼300 μm diameter cylinder spanning 1.288 mm in cortical depth; Lefort et al., 2009; exact size: 1.9 ± 0.1 barrels or 12,300 ± 647 neurons; Fig. 3*C*,*D*). Lesions did not evoke distal microglial or astrocytic reactions (Fig. 3*E*) and did not penetrate the white matter (Fig. 3*F*). Thus, our approach allows for lesions on the scale of individual cortical columns.

### Columnar-scale vS1 lesions impair performance on go/no-go object location discrimination task

**Figure 4.**
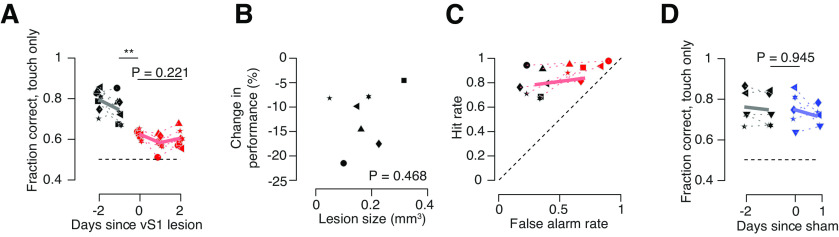
Columnar-scale lesions impair performance on go/no-go location discrimination task. ***A***, Performance before (black) and after (red) the lesion. Solid line, Mean across mice (*n* = 7). Symbols for individual mice are the same as in Figure 3, *D* and *F*. ***B***, Change in performance following lesion as a function of lesion size. *p*-Value, Pearson correlation. ***C***, Change in hit and false alarm rates following lesion. Performance is for day before and last post lesion day. Large symbols, Mean across mice; black, pre lesion; red, post lesion. ***D***, Performance before (black) and after (blue) sham lesion delivered to visual area. Solid line, Mean across mice. **p < 0.01.

We next examined the impact of columnar-scale lesions on performance for the go/no-go object location discrimination task. We recorded high-speed whisker video for at least 2 d before the lesion and 3 d following the lesion (Fig. 3*A*). To assess behavior on trials where the animal was performing object location discrimination, we restricted our analyses to trials on which touch occurred (73.7 ± 2.7% of trials on pre lesion sessions, 71.7 ± 4.2% of trials post lesion, *n* = 7 mice). Following a lesion, performance on touch trials declined from 74.4 ± 2.5% correct to 62.4 ± 0.8% (Fig. 4*A*; *p* = 0.002, paired *t* test comparing day before lesion to day of lesion; *n* = 7 mice). We checked for recovery in the days following the lesion, but performance remained low on the third postlesion session (day 2 post lesion: 60.3 ± 1.6%; *p* = 0.221, lesion day vs day 2 post lesion; *n* = 7). The decline in performance was not sensitive to the size of the lesion (*R* = 0.48, *p* = 0.468, Pearson correlation; Fig. 4*B*). This decline was primarily because of an increase in the fraction of false alarms (from 29.2 ± 3.1% to 70.2 ± 5.6%; *p* < 0.001, paired *t* test comparing day before to the day after lesion; *n* = 7 mice; Fig. 4*C*). Sham lesions of comparable size (0.17 ± 0.13 mm^3^; *n* = 5 mice; *p* = 0.989, unpaired *t* test comparing sham to vS1 lesion volumes; Fig. 3*D*) in visual areas posterior and medial to vS1 performed in a subset of animals did not produce behavioral effects (performance before: 74.5 ± 2.8%; after: 74.8 ± 2.9%; *p* = 0.945; *n* = 5; Fig. 4*D*). Thus, columnar-scale vS1 lesions drive irreversible declines in performance on object location discrimination tasks.

**Figure 5.**
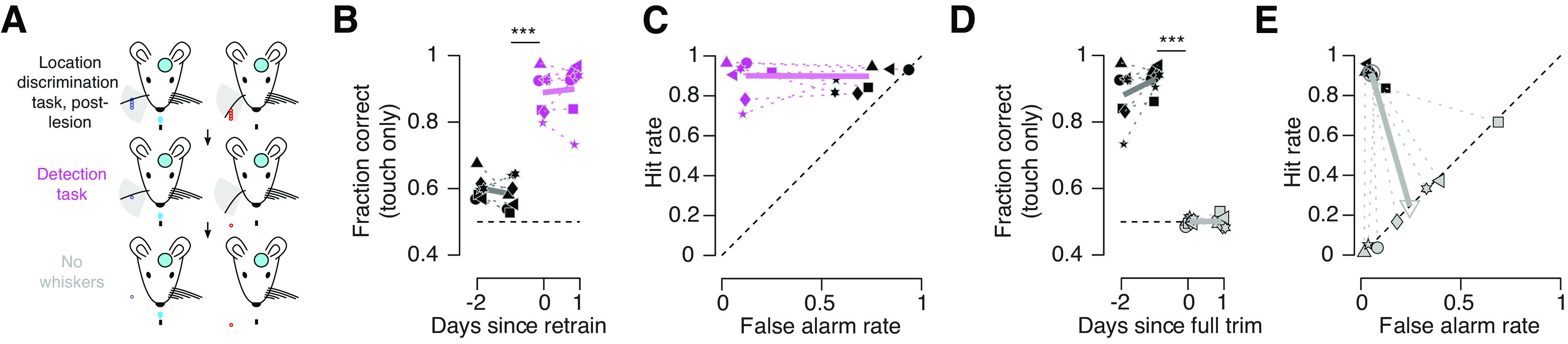
Lesioned animal detection task and post-trim performance. ***A***, Transition from post lesion object location discrimination to detection, and then to full whisker trimming. ***B***, Fraction correct before (black) and after (magenta) transition to detection task. Symbols, Individual mice; thick line, average across mice. ***C***, False alarm and hit rate before (black) and after (magenta) transition to detection task. Thick line, average across mice. ***D***, Fraction correct before (black) and after (gray) removal of whisker. ***E***, False alarm and hit rate before (black) and after (gray) whisker removal. ***p < 0.001.

The increase in false alarm rate on no-go (distal position) trials suggested that post lesion, mice adopted a strategy of licking on object contact regardless of object position and withholding licking when touch did not occur. To test this hypothesis, following the first 3 post lesion days, mice were transitioned to a go/no-go lick-on-touch detection task in which the distal pole positions were completely out of reach and mice simply had to lick on trials where the pole was in reach (Fig. 5*A*). Upon moving lesioned mice to this task, their performance improved from 58.4 ± 1.8% to 88.9 ± 2.5% (Fig. 5*B*; *p* < 0.001, paired *t* test comparing last post lesion location discrimination day to first detection day; *n* = 7 mice). This improvement was mostly because of a decline in false alarm rate (from 71.6 ± 5.1% to 10.0 ± 2.7%; *p* < 0.001; *n* = 7 mice; Fig. 5*C*), implying that lesions had not impaired the capacity of mice to perform at high levels within this go/no-go task structure but instead had interfered with sensory input. In all mice, performance on this detection task exceeded the performance on the final pre lesion object location discrimination task day (*p* < 0.001; *n* = 7), implying that mice performing the object location discrimination task are fundamentally perceptually limited relative to mice performing the detection task.

If mice had indeed adopted a lick-on-touch strategy following the lesion, mice experiencing no touch should withhold licking. To evaluate this possibility, we cut the remaining whisker after 2 days of the detection task and examined the impact on behavior. Performance dropped to chance (performance before trimming, 86.5 ± 4.6%; performance after trimming, 50.0 ± 0.5%; *p* < 0.001; *n* = 7; Fig. 5*D*). All mice adopted a constant lick rate, as evidenced by a matched hit and false alarm rate (Fig. 5*E*), consistent with a guessing strategy. Most mice licked on <25% of trials, substantially less than before trimming, despite their being highly motivated to do so. This is consistent with a lick-on-touch strategy.

Thus, columnar-scale vS1 lesions persistently disrupted performance on an object location discrimination task. Mice adopted a lick-on-touch strategy following the lesion, as demonstrated by their ability to immediately perform a detection task and their near cessation of licking following whisker trimming.

### Columnar-scale lesions do not impact vibrissal kinematics

**Figure 6.**
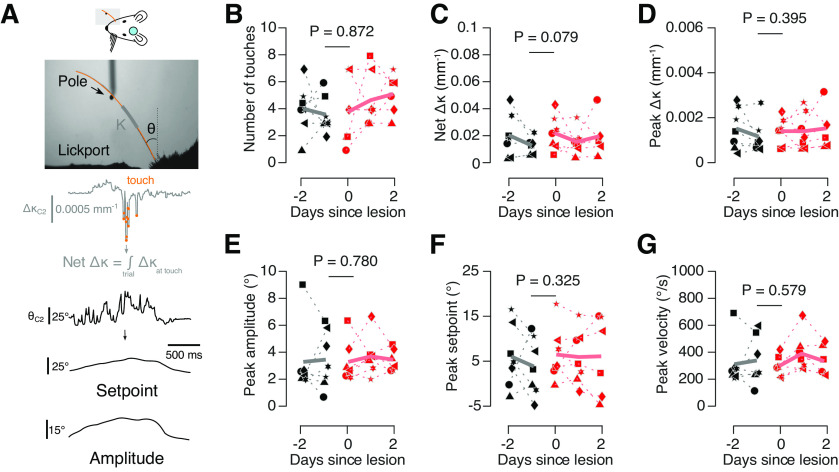
Lesions do not impact vibrissal kinematics. ***A***, Kinematic variables measured. Top, Example whisker video frame, showing curvature (κ, gray) and angle (θ, black) measurement; middle, change in whisker curvature (Δκ, gray; Materials and Methods), with touches overlaid as orange circles, and calculation of net Δκ; bottom, whisker angle over time (θ, black), with whisking setpoint and amplitude obtained from a Hilbert transform (Materials and Methods). ***B***, Number of touches per trial before lick response before (black) and after (red) lesioning in mice performing the go/no-go object location discrimination task. Thick line, Cross-animal mean. *p*-Value provided for paired *t* test comparing pre lesion day to day of lesion (*n* = 7 mice). ***C***, Mean net curvature change (net Δκ), a measure of total touch force over a trial, up to the moment of first lick before (black) and after (red) lesion. ***D***, Peak curvature change (Δκ), a measure for maximum force exerted during touch, before (black) and after (red) lesion. The “peak” was the 99th percentile of values over epochs during which the mouse was touching the pole. ***E***, Peak whisking amplitude (Materials and Methods) before (black) and after (red) lesion. The peak was the 99th percentile of values over epochs during which the pole was in reach but before the first touch. ***F***, Peak whisking setpoint (Materials and Methods) before (black) and after (red) lesion. The peak was the 99th percentile of values over epochs during which the pole was in reach but before the first touch. ***G***, Peak whisker velocity before (black) and after (red) lesion. The peak was the 99th percentile of values over epochs during which the pole was in reach but before the first touch.

Area scale lesions and optogenetic silencing of vS1 alter vibrissal kinematics, with mice exhibiting reduced whisking vigor and lower touch intensities (Hong et al., 2018). Do columnar-scale lesions drive shifts in vibrissal kinematics? To address this question, we compared kinematics before and after lesions (Fig. 6*A*). In mice performing the go/no-go location discrimination task, columnar-scale lesions did not show any effect on the kinematic parameters we measured. Mice made 3.6 ± 1.4 touches per trial before the lesion, and 3.9 ± 2.4 touches per trial after (Fig. 6*B*; *p* = 0.872, paired *t* test; *n* = 7 mice). The intensity of the touches also did not change, quantified using the net curvature change per trial (net Δκ; Fig. 6*C*; before lesion, 0.014 ± 0.006 mm^−1^; after lesion, 0.023 ± 0.013 mm^−1^; *p* = 0.079; Materials and Methods). Peak curvature change was not impacted by lesions (Fig. 6*D*; before lesion, 0.0011 ± 0.0006 mm^−1^; after lesion, 0.0013 ± 0.0005 mm^−1^; *p* = 0.395). Whisking intensity also remained constant (Fig. 6*E–G*; Materials and Methods), quantified using peak whisking amplitude (before lesion, 3.5 ± 2.1°; after lesion, 3.4 ± 1.5°; *p* = 0.780), peak setpoint (before lesion, 4.1 ± 6.4°; after lesion, 6.7 ± 5.9°; *p* = 0.325), and peak velocity (before lesion, 332.6 ± 177.8°/s; after lesion, 291.54 ± 74.3°/s; *p* = 0.579). Thus, whisking kinematics were not altered by columnar-scale lesions, implying that observed behavioral changes were not because of changes in afferent input.

### Columnar-scale lesions interfere weakly and transiently with simple and complex vibrissal object detection tasks

**Figure 7.**
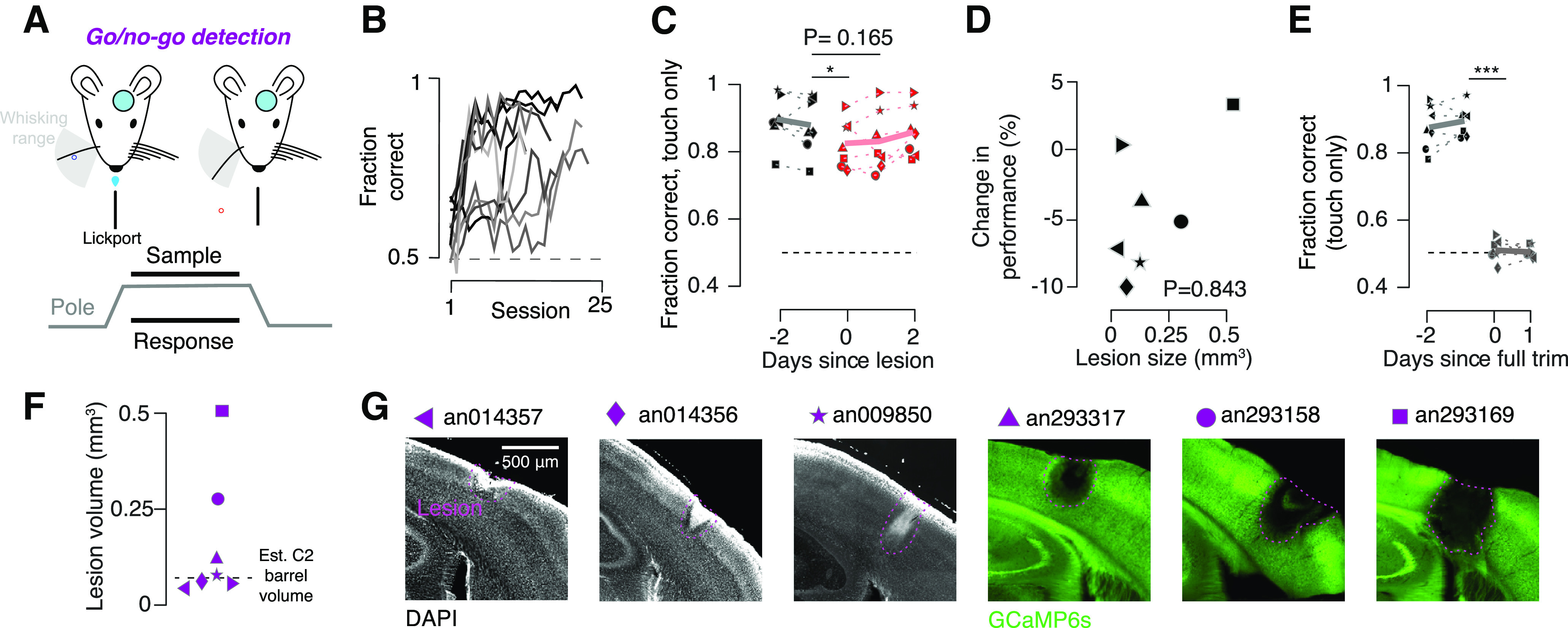
Lesions do not permanently impair performance on go/no-go detection task. ***A***, Task schematic for go/no-go detection task. Top, Mice with a cranial window use a single whisker to localize a pole that appears in either a posterior (blue) or out-of-reach, anterior (red) position. Bottom, Task timing. ***B***, Training progression for all go/no-go detection mice (*n* = 10). ***C***, Performance before (black) and after (red) the lesion; solid line, mean across mice (*n* = 7). *p*-Value provided for paired *t* test comparing pre lesion day to day of lesion. ***D***, Change in performance as a function of lesion size. *p*-Value, Pearson correlation. ***E***, Fraction correct before (black) and after (gray) removal of last remaining whisker. ***F***, Distribution of lesion (purple) volumes. Symbols, individual mice; dashed line, estimated C2 barrel volume. ***G***, Representative slice showing the lesion from go/no-go detection animals. First three slices are DAPI stained, and the last three slices are from transgenic animals expressing GCaMP6s. Wherever possible, the slice shown includes the greatest observed lesion extent. *p < 0.05; ***p < 0.001.

Mice with vS1 lesions show degraded performance on an object location discrimination task but attain high performance on a lick-on-touch detection task (Fig. 5*B*). We therefore asked whether columnar-scale lesions to vS1 would perturb performance in mice trained only on a detection task. Following area-scale vS1 lesions, mice exhibit temporary declines in performance on a go/no-go touch detection task (Hong et al., 2018). We therefore trained a separate cohort of mice on a go/no-go vibrissal object detection task (Fig. 7*A*). Mice learned this task more readily than the discrimination task, reaching above-threshold performance in 3.4 ± 0.5 d (Fig. 7*B*; *n* = 10 mice; *p* < 0.001, *t* test comparing detection and discrimination cohorts). We observed a small decline in performance on the first day following the lesion, despite lesions taking place within an hour of the start of the post lesion behavioral session (performance before lesion, 87.4 ± 2.9%; performance after legion, 82.5 ± 2.4%; *p* = 0.035; *n* = 7 mice; Fig. 7*C*). Lesion size (0.18 ± 0.06 mm^3^; *n* = 7 mice; Fig. 7*F*,*G*) did not predict the effect on performance on the first post lesion day (Fig. 7*D*; *R* = 0.105, *p* = 0.843, Pearson correlation) and did not differ from the location discrimination task (*p* = 0.857, unpaired *t* test comparing go/no-go detection to location discrimination lesion volumes). Behavior quickly recovered to pre lesion levels (*p* = 0.165, *t* test comparing the day before the lesion to the second post lesion day). We confirmed that this was a whisker-dependent behavior by trimming the whisker after 3 post lesion days. Following whisker trimming, mice performed at chance levels (performance before trimming, 88.8 ± 1.6%; performance after trimming, 51.1 ± 1.1%; *p* < 0.001, *n* = 7; Fig. 7*E*).

**Figure 8.**
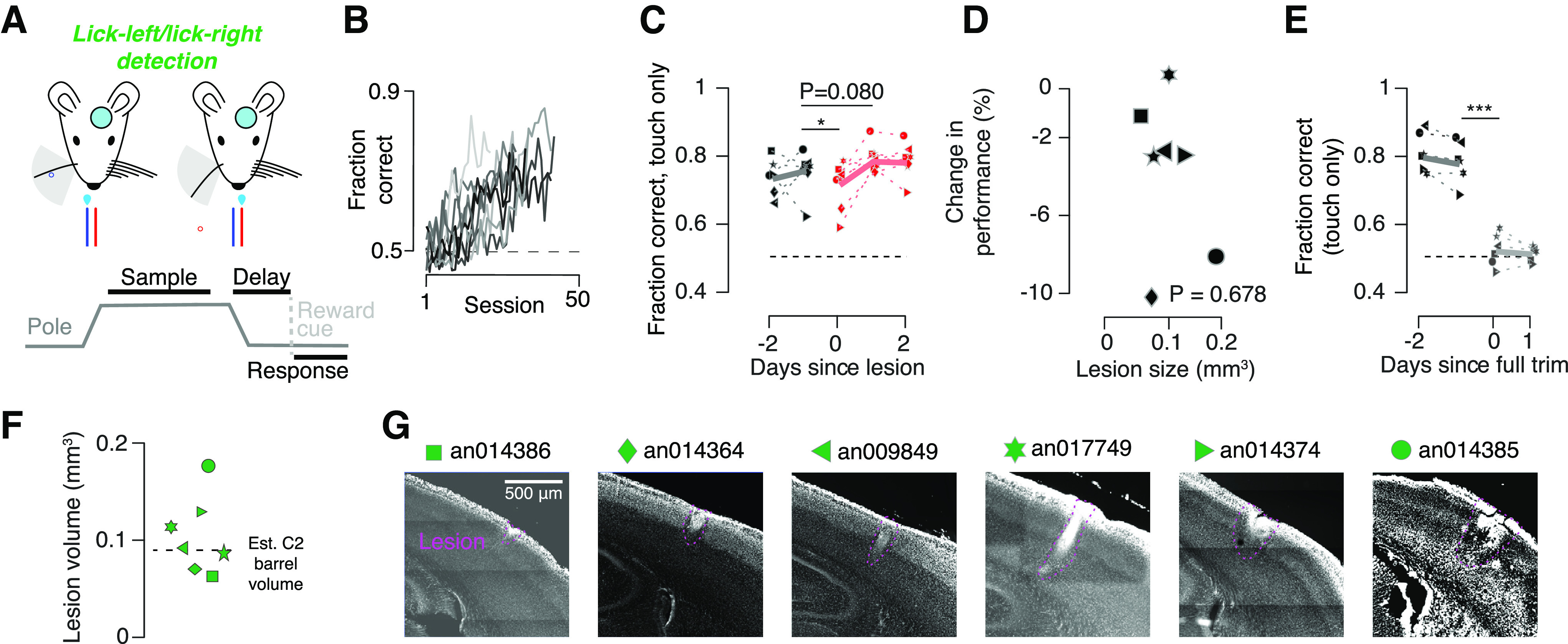
Lesions do not permanently impair performance on lick-left/lick-right detection task. ***A***, Task schematic for lick-left/lick-right (2 lickport) detection task. Top, Mice with a cranial window use a single whisker to localize a pole that appears in either a posterior (blue) or out-of-reach, anterior (red) position, and lick one lickport to report touch and the other to report no touch. Bottom, Task timing. Animals respond after a short delay following the sample period on reward cue. ***B***, Training progression for all lick-left/lick-right detection mice (*n* = 9). ***C***, Performance before (black) and after (red) the lesion. Solid line, mean across mice (*n* = 8). ***D***, Change in performance as a function of lesion size. *p*-Value, Pearson correlation. ***E***, Fraction correct before (black) and after (gray) removal of last remaining whisker. ***F***, Distribution of vS1 lesion (purple) volumes for this task. Symbols, Individual mice; dashed line, estimated C2 barrel volume. ***G***, Representative slice showing the lesion from lick-left/lick-right detection animals. Wherever possible, the slice shown includes the greatest observed lesion extent. *p < 0.05; ***p < 0.001.

Tasks with higher cognitive load are believed to be more dependent on cortical activity than simpler tasks (Stüttgen and Schwarz, 2018). Thus, we examined the impact of columnar-scale lesions on a more complex detection behavior. We trained mice to perform a lick-left/lick-right touch detection behavior that required mice to report touch by licking one of two lickports and the lack of touch by licking the other lickport (Fig. 8*A*). In contrast to the go/no-go behaviors, this task required mice to withhold licking until 0.5 s after the pole became inaccessible, at which point an auditory cue indicated to the animal that it was time to respond. Thus, this task had both two response contingencies and a working memory component. Mice learned this task more slowly than the go/no-go detection task, reaching above-threshold performance in 19.0 ± 2.5 d (Fig. 8*B*; *n* = 9 mice; *p* < 0.001, *t* test comparing go/no-go and lick-left/lick-right detection cohorts). Despite the greater difficulty in learning this task, mice still showed only a very weakly significant performance decline immediately following a columnar-scale lesion in vS1, which recovered by the next day (performance before lesion, 73.9 ± 2.2%; performance after lesion, 69.8 ± 2.5%; *p* = 0.039; *n* = 7; performance on second post lesion day, 77.7 ± 1.4%, *p* = 0.080, *n* = 7; Fig. 8*C*). Lesion size (0.10 ± 0.01 mm^3^; *n* = 7 mice; Fig. 8*F*,*G*) did not correlate with performance (Fig. 8*D*; R = 0.193, *p* = 0.678, Pearson correlation) and did not differ from the location discrimination task (*p* = 0.090, unpaired *t* test comparing lick-left/lick-right detection to location discrimination lesion volumes). Performance fell to chance levels following whisker trimming (performance before trimming, 77.2 ± 2.2%; performance after trimming, 52.7 ± 1.7%; *p* < 0.001; *n* = 7; Fig. 8*E*). Thus, vS1 was still not necessary for task performance on a detection task, even after adding a working memory component and a second response contingency.

## Discussion

We tested the behavioral role of vS1 columns in a range of vibrissa-dependent tasks and found that lesioning one to two barrels in vS1 degraded performance when the mouse had to report whether it touched a proximal or distal object. Lesions only slightly degraded performance when the mouse had to report whether it touched an object or not. The deficit in the location discrimination task performance did not recover for several days following the lesion. In contrast, detection performance recovered by the second post lesion day, even in a task with a working memory component. Thus, neurons within the principal whisker column in vS1 are necessary for vibrissal object location discrimination, but not for touch detection.

Identifying neurons that underpin perception ultimately requires identifying structures whose removal permanently perturbs behavior. Transient inactivation of a structure can often lead to a behavioral deficit, even if permanent removal of that structure does not influence behavior or produces rapid recovery (Otchy et al., 2015; Hong et al., 2018). Finding structures that are indispensable to specific behaviors thus requires permanent inactivation followed by longitudinal observation (LaMotte and Mountcastle, 1979). Lesions are well suited for this purpose. Our work presents a spatially precise lesioning approach which allows us to measure the behavioral role of small cortical structures and track recovery over time. Our use of this lesioning method to target individual vS1 barrels demonstrates that small cortical volumes play a crucial role in the perception of object location, as the behavioral impact of permanent, columnar-scale lesions persisted for several days (Wolff and Ölveczky, 2018).

Vibrissal S1 has been implicated in many whisker behaviors using a range of inactivation approaches. These approaches, both transient and permanent, often inactivate not only vS1 but also adjacent structures. In the case of transient inactivation, the radius of effect is rarely measured, but when it is, it usually extends to at least 1 mm (Krupa et al., 1999; Long and Fee, 2008; Hong et al., 2018; Li et al., 2019). Spatially extensive inactivation can impact adjacent structures, which can lead to misattribution of the behavioral role. For example, lesions of vS1 that extend to striatum can produce permanent performance degradation in touch detection tasks where lesions confined to vS1 produce behavioral recovery (Hong et al., 2018). Area-scale lesions of vS1 have led to degraded performance in tasks requiring discrimination of aperture size (Krupa et al., 2001), object location (O’Connor et al., 2010), distance (Hutson and Masterton, 1986), and texture (Guic-Robles et al., 1992). In contrast, whisker touch detection tasks show either no sensitivity to lesions (Hutson and Masterton, 1986) or transient sensitivity (Hong et al., 2018), with rapid recovery back to pre lesion performance. Local pharmacological inactivation degrades performance in a single-vibrissa object location discrimination task (O’Connor et al., 2010) as well as tasks that require comparison of vibrissal vibration across two sides of the head or detection of vibration on one side (Miyashita and Feldman, 2013). Optogenetic silencing of vS1 interferes with object location discrimination (Guo et al., 2014b) and object detection (Sachidhanandam et al., 2013; Hong et al., 2018). Spatial dissociation of function in auditory cortex demonstrates that specific regions subserve specific functions (Lomber and Malhotra, 2008). Consequently, whether these tasks require large portions of vS1, surrounding areas that are likely impacted by past inactivation approaches, or just the relevant whisker barrels, remains unclear. Our work shows that relatively small populations of neurons can underpin specific behavioral functions.

Gap crossing with a single whisker, which is impeded following large-scale vS1 lesions (Hutson and Masterton, 1986), is susceptible to microstrokes on the scale of a single barrel (Shih et al., 2013). These strokes are generated by occluding blood vessels either via laser illumination of a sensitizer or via amplified laser pulses. Columnar-scale lesions are comparable in size to microstrokes but are produced without the need for targeting blood vessels or the use of special reagents. Both approaches demonstrate that relatively small (10,000–20,000) populations of neurons can contribute substantially to behavior, raising the intriguing possibility that an even smaller subset of these neurons may act as a bottleneck in the generation of perception. In the case of gap crossing, it is unclear whether performance would have recovered after several days as performance was only evaluated in a single post lesion session (Shih et al., 2013).

Primary sensory cortices are thought to be more important for behaviors involving more complex stimuli and higher cognitive loads. In the auditory cortex, many discrimination behaviors require cortex (Lomber and Malhotra, 2008; Slonina et al., 2022). At the same time, simple auditory features such as frequency can be discriminated even without cortex (Ohl et al., 1999). Lesions to V1 in humans result in “blindsight”—the ability to perform certain vision-dependent tasks despite the lack of conscious visual awareness (Leopold, 2012). Complex visual tasks, however, can no longer be performed. In vS1, performance on tasks where a relatively strong stimulus (e.g., active touch) needs to be detected recovers or does not degrade after inactivation (Hutson and Masterton, 1986; Hong et al., 2018), while performance on object location discrimination (O’Connor et al., 2010) or gap crossing (Hutson and Masterton, 1986) tasks is affected. Tasks with a higher cognitive load are also thought to be more dependent on activity from primary sensory cortices than those with a lower cognitive load (Stüttgen and Schwarz, 2018). We find that individual vS1 barrels are necessary for object location discrimination but are only transiently necessary in both our go/no-go detection task and the more complex lick-left/lick-right delayed response detection task. Thus, stimulus complexity but not cognitive load predicts the importance of individual vS1 barrels.

What is the minimal set of neurons needed for object location discrimination in vS1? In layer (L) 2/3, ∼10% of neurons show robust responses to touch (Crochet et al., 2011; Peron et al., 2015). L4 excitatory neurons produce fewer spikes, on average, than L2/3 neurons, and L5 neurons produce comparable spike counts (Yu et al., 2019). This suggests that a sparse minority of the ∼6000 excitatory neurons in a barrel (Lefort et al., 2009) contribute to the touch response. It is thus possible that several hundred neurons act as a perceptual bottleneck in vS1 for single whisker object location discrimination. In contrast to columnar-scale lesions, however, cellular resolution lesions in vS1 targeting tens of neurons have not produced behavioral effects (Peron et al., 2020). This result is unsurprising given that those animals were performing a detection task, which we have here shown to be insensitive to vS1 lesions. Cellular resolution lesion experiments in mice performing object location discrimination tasks will thus be crucial in determining whether small populations of neurons constitute a perceptual bottleneck in vS1.

In vS1, mice can readily detect the activation of arbitrary groups of tens of neurons (Dalgleish et al., 2020). Even single neurons can drive behavioral report (Houweling and Brecht, 2008); among excitatory neurons in vS1, touch-sensitive pyramidal neurons show the greatest ability to drive a single-neuron perceptual report (Tanke et al., 2018). In mouse primary visual cortex, activating a handful of neurons can perturb discrimination between vertical and horizontal gratings (Carrillo-Reid et al., 2019; Marshel et al., 2019). These experiments do not, however, demonstrate that naturalistic percepts only require tens of neurons. The introduced activity is likely amplified via the strong recurrence present in cortex (Douglas and Martin, 2007; London et al., 2010), enabling perceptual detection. Consistent with this argument, in tongue premotor cortex, activating ∼10 neurons can perturb the chosen licking direction (Daie et al., 2021), but silencing does not exert an effect unless hundreds of thousands of neurons are inactivated (Guo et al., 2014a). Moreover, adding activity to hundreds of L4 vS1 neurons only partially increases the report of touch (O’Connor et al., 2013). Thus, while gain-of-function experiments are crucial to our understanding of the neural basis of perception, loss-of-function experiments are needed to establish that a given population plays a role in a naturalistic behavior. Our experiments establish a ceiling of ∼10,000 excitatory neurons as a perceptual bottleneck for the discrimination of naturalistic touch.

In sum, we present a spatially precise lesioning method that can be used to study the role of cortical areas across a variety of task types. Applying this method, we find that individual vS1 barrel columns contribute to single-whisker vibrissal object location discrimination. At the same time, object detection using active whisking is not dependent on individual barrel columns. Thus, small (∼10,000) populations of primary sensory cortical neurons can contribute to specific behaviors.

## Synthesis

Reviewing Editor: Miriam Goodman, Stanford University Decisions are customarily a result of the Reviewing Editor and the peer reviewers coming together and discussing their recommendations until a consensus is reached. When revisions are invited, a fact-based synthesis statement explaining their decision and outlining what is needed to prepare a revision will be listed below. The following reviewer(s) agreed to reveal their identity: NONE. Note: If this manuscript was transferred from JNeurosci and a decision was made to accept the manuscript without peer review, a brief statement to this effect will instead be what is listed below.

Editor’s note: The authors have been very responsive to reviewer’s comments, addressing queries and concerns regarding interpretation and comparison to prior work. In my reading, I note the following suggestions for improvement and minor corrections:

1. Statistical reporting: For summary data provided in the text please include the nature of the error provided and the number of measurements used to compute average values in every instance OR state the nature of the error used in the Statistical Methods section. As an example, the following text (p. 16) provides the number of animals tested but not the nature of the error: “To assess behavior on trials where the animal was performing object location discrimination, we restricted our analyses to trials on which touch occurred (73.7 {plus minus} 2.7% of trials on pre-lesion sessions, 71.7 {plus minus} 4.2% of trials post-lesion, n = 7 mice).”

2. The correct nomenclature for C57Black mice is “C57BL/6J” not “C57/BL6J” https://www.jax.org/strain/000664
